# Can artificial intelligence in orthopantomography advance dental diagnostics through automated image analysis?

**DOI:** 10.3389/fradi.2026.1701356

**Published:** 2026-02-19

**Authors:** Dan Adrian Lutescu, Claudiu Constantin Manole, Ana Maria Cristina Țâncu, Radu Ilinca, Reinhard Chun Wang Chau, Szabolcs Felszeghy, Andreea Cristiana Didilescu

**Affiliations:** 1 Department of Medical Informatics and Biostatistics, Faculty of Dentistry, “Carol Davila” University of Medicine and Pharmacy, Bucharest, Romania; 2 Department of Biophysics, Faculty of Dentistry, “Carol Davila” University of Medicine and Pharmacy, Bucharest, Romania; 3 Department of Prosthodontics, Faculty of Dentistry, “Carol Davila” University of Medicine and Pharmacy, Bucharest, Romania; 4 Faculty of Dentistry, The University of Hong Kong, Hong Kong, Hong Kong SAR, China; 5 Institute of Dentistry, School of Medicine, University of Eastern Finland, Kuopio, Finland; 6 Department of Embryology and Microbiology, Faculty of Dentistry, “Carol Davila” University of Medicine and Pharmacy, Bucharest, Romania

**Keywords:** artificial intelligence, digital dentistry, image processing, orthopantomograms, teeth detection

## Abstract

Artificial Intelligence (AI) is rapidly transforming dental education and clinical practice, as deep learning—especially convolutional neural networks—brings unprecedented accuracy to interpreting orthopantomograms (OPGs). This review illuminates the cutting-edge frontiers of AI-driven dental imaging, tracing how recent breakthroughs are transforming the detection, classification, and segmentation of complex dental anatomy and pathology. Notably, state-of-the-art AI models have reached remarkably high accuracy in tooth identification, while commercial solutions demonstrate promising—though variable—performance in diagnosing complex conditions, such as the adequacy of endodontic procedures. Yet, bringing AI into routine dental care remains fraught with obstacles — demanding vast annotated datasets, coping with population variability, and confronting persistent medicolegal and trust concerns. Growing collaborations between regulators and professional bodies in the United States and European Union are now shaping ethical and legal frameworks to guide its safe use. This narrative review goes beyond summarizing technological progress in AI-driven dental radiology — it uniquely integrates diagnostic breakthroughs with the rapidly evolving regulatory and ethical landscape. By bridging innovation with implementation, it offers educators, practitioners, and learners a forward-looking roadmap that positions AI not as a distant promise, but as a transformative force already reshaping the future of dental diagnostics and training.

## 1 Introduction

Artificial Intelligence (AI) is a machine-based system that can, for a given set of human-defined objectives, make predictions, recommendations, or decisions, thereby performing tasks typically carried out by humans (1).

AI has demonstrated significant advances across multiple medical fields, supporting complex tasks such as image segmentation, disease detection, and outcome prediction. AI is used with results comparable to expert clinicians in fields such as oncology [e.g., the detection of pancreatic tumors from MRI scans (2)], ophthalmology [e.g., mapping the retinal nerve fiber damage (3)], neurology [e.g., predicting surgical outcomes in patients with temporal lobe epilepsy (TLE) (4)], and colorectal cancer detection (5).

The achievements underscore the growing reliability of AI in medical imaging, in general, and establishes a foundation for its application in dental diagnostics, particularly in the analysis of orthopantomograms (OPGs), also known as panoramic radiographs.In dentistry, AI has shown comparable performance to specialists in interpreting panoramic radiographs and intraoral images. Models trained on annotated OPGs achieved accuracy in tooth detection and numbering, highlighting their potential to support clinical workflows and to automate diagnostic tasks (6). This is confirmed by other studies, which show that AI systems have matched clinicians in diagnosing periodontal conditions such as attachment loss and gingival inflammation (7, 8). Growing precision and versatility of AI models have been achieved in dental imaging. AI has reached over 89% accuracy in predicting third molar eruption status (9) and has been successfully applied to assess the difficulty of mandibular third molar extractions, supporting clinical decision-making (10). These applications demonstrate the utility of OPGs as a foundational imaging source for AI-based diagnostic tools (11).

Such tools can instantly detect key anatomical landmarks and assess procedural complexity, assisting clinicians during preoperative planning (12) and improving the safety and predictability of interventions such as extractions or implant placement (13).

AI is also being explored as a means to predict disease progression, allowing for more personalized and proactive dental care (14). By processing large volumes of clinical data, AI models can identify risk factors and anticipate treatment needs, facilitating timely interventions and long-term monitoring (15). AI-assisted diagnostic processes can be highly accessible. AI diagnostics can be made with smartphone devices alone, such as the one presented in a validated study of the gingivitis detection by GumAI mobile test tool (16). These predictive tools are especially relevant in complex cases, where clinical decisions depend on a broad set of interrelated parameters.

AI systems are becoming more sophisticated in pediatric dentistry and preventive care (17), particularly in monitoring oral hygiene (18) or detecting early signs of pathology (19). Their capacity to offer consistent evaluations across diverse patient groups makes them valuable allies in reducing diagnostic variability and promoting equitable access to care.

AI algorithms such as Convolutional Neural Networks (CNN) and their derivatives —including You Only Look Once (YOLO) and the Segment Anything Model (SAM)— have driven most recent advances in dental imaging. Meanwhile traditional machine-learning frameworks such as Support Vector Machines (SVM) and Random Forest have been used as comparison benchmarks.

This narrative review addresses the need for a structured synthesis of the AI achievements towards a better understanding of AI's clinical relevance, limitations, and potential for integration into routine workflows. For dental educators and learners, the integration of AI heralds a paradigm shift in training and curriculum design. Beyond augmenting diagnostic accuracy, AI may offer powerful tools for teaching anatomical variation, simulating complex cases, and fostering critical engagement with emerging technologies. By addressing both technological progress and educational adaptation, this review equips stakeholders with the insights needed to prepare the next generation of dentists for an AI-driven future.

The literature search was mainly conducted in PubMed and Scopus databases by identifyng studies published between 2020 and 2025 using the keywords artificial intelligence, dentistry, panoramic radiograph, tooth detection, and diagnostic accuracy. For a broader perspective, Google Scholar was also used as full text search engine. By analyzing recent advances, performance benchmarks, and practical challenges — including regulatory, ethical, and technical aspects — this work provides an overview of the AI state of the art in the field of dental imaging, with a specific focus on OPGs.

## 2 AI in dental imaging diagnostics

### 2.1 Increased diagnostic accuracy: AI reduces human errors and interobserver variability

Artificial intelligence has demonstrated increasing value in improving diagnostic accuracy within dentistry, primarily by reducing human error and interobserver variability. A detection system evaluated for identifying periapical lesions on periapical radiographs, using CBCT as the reference standard, analyzed 1,040 teeth from 52 patients and showed a diagnostic accuracy of 88.2%, with sensitivity and specificity of 91.5% and 83.3%, respectively. Notably, agreement between the AI and expert consensus (Fleiss' kappa = 0.83) was higher than that among the human observers (kappa = 0.71), highlighting AI's capacity to provide consistent, expert-level interpretation (20). Similar results were found in periodontal diagnostics, where an AI-powered system outperformed or matched the accuracy of 60 dentists — including general practitioners, periodontists, and specialists — when analyzing six clinical features on panoramic radiographs; it showed the highest agreement with the gold standard in detecting bone loss (kappa = 0.76), while general dentists exhibited lower consistency (kappa = 0.57) (7). Further supporting this trend, the AI platform Diagnocat achieved 90.72% accuracy and an F1 score of 95.12% in detecting endodontic fillings from panoramic images of 55 patients, though its performance dropped significantly in qualitative assessments such as detecting short fillings (8.33%) and voids (14.29%), suggesting that while effective in recognizing treated teeth, its finer evaluations require further refinement (21). In a broader diagnostic context, an AI-based software analyzing 600 panoramic radiographs from patients with permanent dentition achieved 90% sensitivity, 85% specificity, 88% accuracy, and 87% precision in identifying dental anomalies, reinforcing its potential to reduce diagnostic variability and clinician-dependent inconsistencies (22). Even in pediatric cases, an AI algorithm applied to 35 panoramic images of developing dentition showed specificity and accuracy above 85% in detecting caries, fillings, and missing teeth, though with slightly lower precision, indicating the promise of AI in pediatric diagnostics and the continued need for improvement to ensure consistent clinical performance (23).

### 2.2 Efficiency and speed: AI can process large volumes of images quickly, optimizing clinical workflow

AI systems are increasingly demonstrating their ability to handle large datasets rapidly and accurately, offering tangible improvements in clinical efficiency and workflow optimization. A notable example comes from a cross-sectional study involving 11,380 patients from Kielce, Poland, where an AI system trained on over 250,000 dental panoramic radiographs was employed to evaluate oral health. The algorithm automatically assessed multiple conditions, including caries, fillings, root canal treatments, implants, crowns, endodontic lesions, and missing teeth. It identified dental caries in 59.3% of patients, fillings in 76.2%, missing teeth in 77.5%, root canal treatments in 16.5%, implants in 4.1%, and crowns in 2.6%, demonstrating the system's capacity to efficiently process high-volume data and produce standardized outputs useful for large-scale treatment planning and monitoring (24).

In a different application focused on dental plaque detection, an AI system was developed using deep learning techniques to classify plaque stages in clinical photographs. The study involved 531 color images (RGB) from 177 individuals, captured with various mobile devices. Each image was treated with a disclosing gel to enhance visibility of plaque, then preprocessed for lighting and color normalization. YOLOv9, YOLOv10, and YOLOv11 models were trained to detect three distinct stages of plaque: new, mature, and over-mature. Among them, YOLOv11 m achieved the highest mean Average Precision (mAP) at 0.713, demonstrating strong detection performance, particularly for over-mature plaque. The use of the O’Leary index further revealed that over 50% of the study population exhibited severe plaque levels, highlighting the system's potential to support early diagnosis and preventive care, while reducing the diagnostic burden in clinical workflows (25).

The ability of AI to accelerate diagnostic processes was also demonstrated in a study employing a deep learning model based on ResNet architecture to detect osteoporosis-related changes in OPGs. The dataset included 340 OPGs, evenly split between osteoporotic and non-osteoporotic patients, diagnosed via DEXA. The system achieved an overall accuracy of 92.6%, with sensitivity and specificity exceeding 91%, and processed each image in under one second. Grad-CAM visualizations were integrated to support clinical relevance and interpretability. These results illustrate the model's suitability for opportunistic screening of osteoporosis during routine dental examinations, enabling faster decision-making and improved workflow (26).

In another study, a two-stage deep learning framework was developed to detect periapical lesions (PALs) on panoramic radiographs. Using 713 OPGs and 18,618 periapical root areas, the system achieved an overall accuracy of 84.6%, sensitivity of 72.2%, and specificity of 85.6%, demonstrating the feasibility of automated detection of periapical pathologies in clinical workflows (20).

### 2.3 The general limitations and constraints of artificial intelligence

Despite the promising diagnostic capabilities of AI, several studies have highlighted key challenges and limitations that impact its integration into routine dental practice. Workload concerns were highlighted in a qualitative study involving 8 dentists and 5 patients. The study explored real-world barriers to adoption, revealing that generating structured diagnostic reports may take up to 15 min per panoramic radiograph, often resulting in incomplete documentation due to time pressure (27).

Another study provided diagnostic accuracy without AI averaged at 73.6%, while with AI, it increased to 85.8%. However, interobserver variability remained, and the model showed limitations in areas with overlapping anatomical structures or low image quality, indicating that although AI improves performance, it remains dependent on image clarity and anatomical context, which limits its standalone reliability (8). In the field of periodontal diagnostics, AI has also been shown to perform comparably to clinicians. In a study comparing AI with 60 dentists — including general practitioners, periodontists, and specialists — across six clinical features assessed on panoramic radiographs, the AI system demonstrated equal or superior accuracy in several parameters, particularly bone loss detection (kappa = 0.76 vs. 0.57 for general dentists). Nonetheless, variability in more complex assessments highlighted the need for further refinement and validation before widespread clinical adoption (7).

## 3 AI applications in OPG analyses

### 3.1 Detection of dental pathologies: caries, fractures, bone lesions, periodontal diseases

AI has shown considerable promise in detecting a wide range of dental pathologies, including caries, periapical lesions, fractures, and bone-related conditions, although challenges in generalizability and precision remain. A YOLOv8 deep learning model trained on 1,628 panoramic radiographs was used to detect periapical lesions, root fragments, impacted teeth, and prosthetic elements. Internal validation yielded high precision (>0.90) and recall (>0.80) for implants, endodontic treatments, and surgical devices; however, performance dropped for caries and periapical lesion detection. Furthermore, during external validation across three clinical centers on 180 images, model accuracy declined significantly due to differences in acquisition protocols and image quality, underscoring the need for diverse, well-annotated datasets to support real-world implementation (28). In another study, U-Net, U-Net++, and U-Net3 + architectures were tested on the Detection, Numbering, and Segmentation (DNS) dataset to detect dental cavities. After annotating 510 images and expanding the dataset to 4,296 through data augmentation, U-Net3 + emerged as the top performer, achieving 95% accuracy due to its use of full-scale skip connections and deep supervision. However, variable image quality and artifacts highlighted persistent data quality issues that must be addressed to ensure diagnostic robustness in AI-based system (29). Similarly, a two-stage CNN was developed to detect periapical lesions across 713 panoramic radiographs, with 18,618 periapical root areas annotated as healthy or pathological. The first stage, using Faster R-CNN, achieved an average precision of 74.95% for localizing regions of interest, while the second stage, using an Inception v3 classifier, reached 84% accuracy, 81% sensitivity, and 86% specificity. When both stages were integrated, the combined model achieved 84.6% accuracy, 72.2% sensitivity, and 85.6% specificity — results that support the utility of layered detection approaches for enhancing the accuracy of periapical lesion identification (20).

In pediatric diagnostics, an AI algorithm was tested on 35 OPGs selected from over 1,000 images of developing dentition from patients in Kielce, Poland. The model demonstrated high specificity and accuracy (both above 85%) in detecting caries, fillings, and missing teeth, but lower precision indicated the need for further refinement before reliable clinical adoption in younger populations (23).

AI has also been applied in the detection of systemic bone conditions such as osteoporosis from dental radiographs. In one study, 348 panoramic radiographs were used to train and test a combined model employing YOLOv8 for region detection and EfficientNet for classification. The model achieved 0.83 sensitivity, an F1-score of 0.53, and an AUC of 0.76. Detection varied across mandibular zones, with the angulus region yielding the lowest sensitivity (0.66) and the mental foramen the highest (0.80), highlighting that regional anatomical variation and methodological rigor are critical for accurate AI-assisted osteoporosis screening in dental practice (26).

### 3.2 Segmentation and classification of dental structures: automated identification of teeth, roots, and bone structures

**Figure 1:**
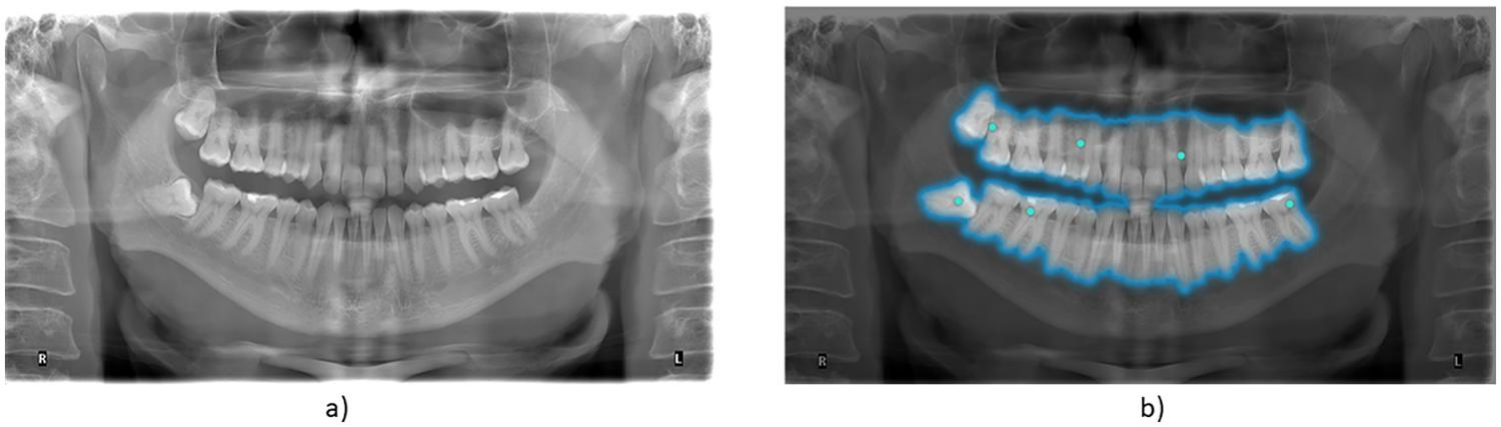
An example orthopantomogram (OPG) shown without **(a)** and with **(b)** teeth partitioning by AI segmentation (SAM2 algorithm). The segmentation in Figure 1b was obtained with tools available at https://github.com/facebookresearch/segment-anything, licensed under Apache License, Version 2.0.

With the aim of increasing the ease of processing and to focus on more meaningful areas of the radiographs, sometimes the images are partitioned in regions of interest by a process called segmentation. An example of segmentation is shown in Figure 1. With the optimized images, the algorithm is subjected to a training period, also called learning. During the training period, the image sets are processed many times, until the desired results are obtained. A complete pass through the image set is called an epoch. The segmentation and the training are the steps involved in preparing the AI for the classification of dental images.

Automated segmentation and classification of dental structures such as teeth, roots, and restorations play a critical role in streamlining diagnosis, treatment planning, and longitudinal monitoring in dental practice, and deep learning-based models have demonstrated high potential in this domain. A study employing the Mask Region-based Convolutional Neural Network (Mask R-CNN) achieved precise instance segmentation of individual teeth using 1,000 panoramic x-rays from the Tufts Dental Database, divided into 760 training images, 190 for validation, and 70 for testing. The model, trained over 300 epochs with a batch size of 10 and a base learning rate of 0.001, reached a mean mAP of 92.49% and an accuracy of 100% in detecting individual teeth, showcasing its ability to precisely delineate dental structures and improve diagnostic reliability (30). In a related effort, the Squeeze and Excitation Inception Block-based Encoder–Decoder (SE-IB-ED) network was introduced for tooth segmentation and numbering, integrating the InceptionV3 encoder with a custom decoder featuring pointwise convolution and attention mechanisms. Trained on 313 panoramic radiographs annotated using the Fédération Dentaire Internationale (FDI) system, the model achieved an F1-score of 92.65%, mean Intersection over Union (mIoU) of 86.38%, accuracy of 92.84%, precision of 92.49%, and an outstanding recall of 99.92%, confirming its precision and robustness in identifying and classifying individual teeth for structured dental charting and planning (6).

Beyond teeth segmentation, a U-Net convolutional neural network was applied to identify and segment dental restorations — including fillings, crowns, and root canal fillings — from 1,781 annotated panoramic radiographs. To address the challenges posed by varying shapes and sizes of restorations, a tiling strategy was employed, segmenting each image into 6, 10, or 20 rectangular sections. The model trained on full images achieved an F1-score of 0.70, while those using 6, 10, and 20 tiles reached progressively higher F1-scores of 0.83, 0.92, and 0.95. The detection of root canal fillings, often small and infrequent, improved by 294% using the tiling method, demonstrating that localized processing significantly enhances AI segmentation performance for fine-grained features (31). Additionally, a comparative study evaluated four deep learning models—U-Net, DCU-Net, DoubleU-Net, and Nano-Net—on a dataset of 1,500 panoramic radiographs, with and without data augmentation. DoubleU-Net yielded the highest accuracy at 96.591% and a Dice coefficient of 92.886% when data augmentation was applied. Notably, Nano-Net, with only 235,425 trainable parameters, achieved comparable results, suggesting it as a resource-efficient option for clinical environments with limited computational capacity (32).

Another model based on the Mask R-CNN architecture was developed to detect the presence or absence of teeth in panoramic radiographs using a dataset of 1,000 images (760 for training, 190 for validation, 50 for testing). Trained under the same hyperparameters—300 epochs, batch size 10, learning rate 0.001—the model achieved 100% accuracy, further reinforcing the reliability of deep learning in basic tooth classification tasks, essential for both diagnostics and automated dental record generation (33).

Together, these findings illustrate the high precision, adaptability, and clinical relevance of AI-based segmentation and classification models in dental imaging, supporting automation and standardization of diagnostic workflows across a wide range of dental structures.

### 3.3 Use of convolutional neural networks (CNNs): the most effective models for panoramic image analysis

**Figure 2:**
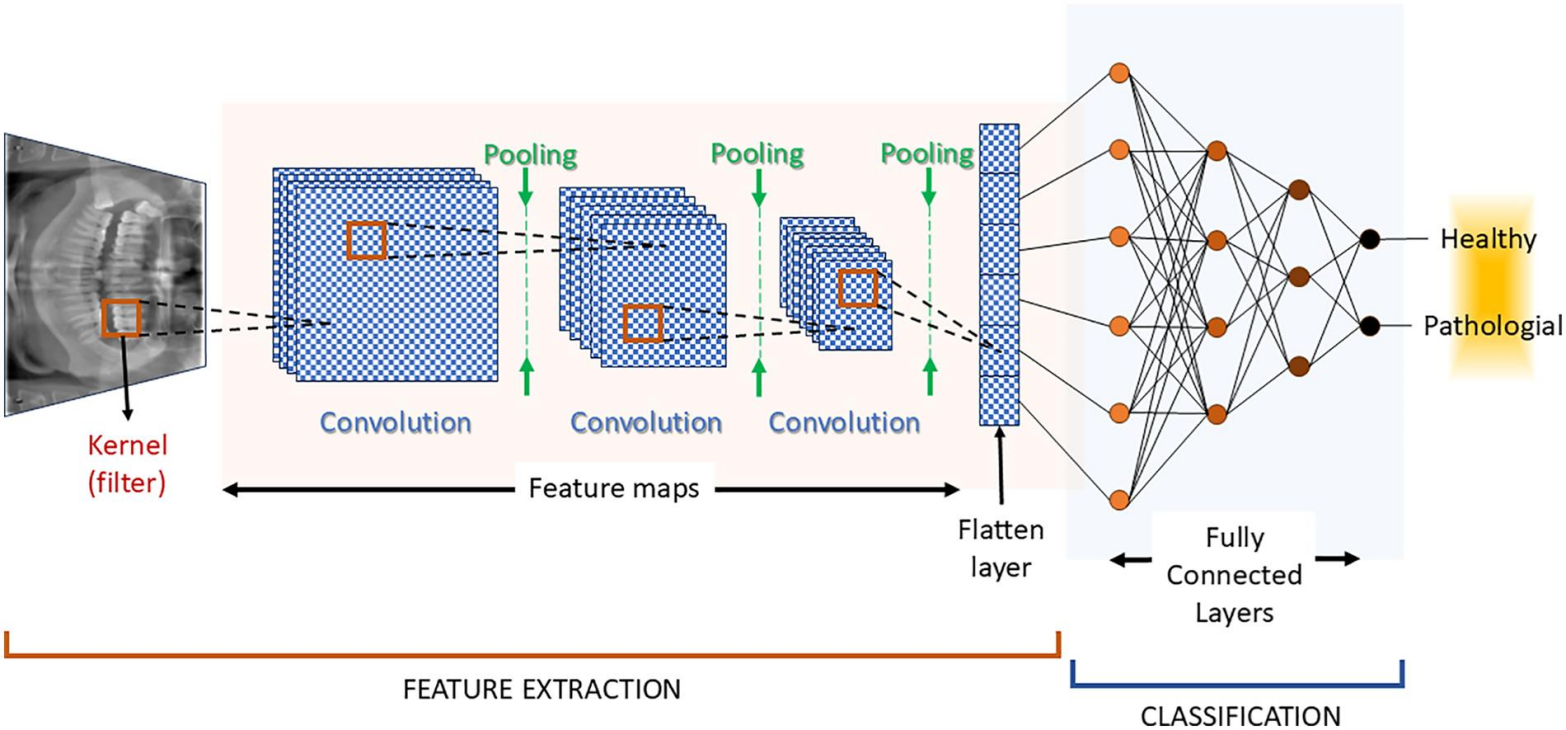
The representation of a typical convolutional neuronal network (CNN) architecture.

CNNs have become a cornerstone in the analysis of panoramic dental radiographs due to their capacity to extract complex features and deliver highly accurate diagnostic outcomes. The inputs on which the CNN is trained are the radiographic images. The CNN architecture can be structured based on three processes: i) convolution process, ii) pooling process and iii) a fully connected neuronal layer weighing. The schematic representation can be seen in Figure 2. The convolution is the process by which two signals are combined in order to obtain a third one. The two signals are the original data (either the image, or another layer of data from the network topology) and a filter, also called kernel. The convolution's role is to detect low-level features from the input (34). A pooling process (35) then reduces the dimensionality by aggregating the data obtained after the convolution. An image contains a relatively large number of pixels, which translates to a large data set that is required to be processed. The convolution and the pooling help reduce the computation burden (36). Successive convolution and pooling steps are repeated (see Figure 2), until a single data layer is obtained, called the flattened layer. The fully connected layers (also called the dense layers) make the final decision, providing the classification required for the output. In Figure 2, there is only a binary output: healthy or pathological. When there are more outputs (e.g., the type of teeth), after the fully connected layers, a probabilistic distribution can be used, such as Softmax (34).

A study evaluating skeletal growth maturation employed CNNs to analyze 1,200 cephalometric radiographs and 1,200 OPGs, using cervical vertebral maturation (CVM) and calcification of the lower second molar as biological indicators. Each image was classified according to the cervical vertebral maturation index (CVMI), with the model achieving 98% accuracy in predicting skeletal maturity in males based on CVM, and comparably high accuracy in females using molar calcification stages, confirming CNN-based multiclass classification as a robust method for skeletal development assessment (37).

Building on the strength of CNNs, a hybrid model combining YOLO and RT-DETR (Real-Time Detection Transformer) was introduced to detect impacted teeth in 407 labeled panoramic radiographs, achieving a mAP of 98.3% and a F1 score of 96%, suggesting that integrating CNNs with transformer-based architectures can further enhance diagnostic accuracy and efficiency in complex localization tasks (38). In another application, YOLO11 sub-models were trained on 2,000 annotated panoramic radiographs to predict the difficulty of mandibular third molar extractions using the Pederson Index, which incorporates Winter's classification (angulation) and Pell and Gregory's criteria (ramus relationship and depth). The nano sub-model attained a mAP of 0.975 for Winter's classification and 0.965 for class, while the medium model reached 0.989 for level. The full system demonstrated a precision of 97.00%, recall of 94.55%, and F1 score of 95.76%, confirming its high reliability in evaluating extraction complexity and its utility in clinical decision-making (10).

CNNs have also been employed in image enhancement through deep learning-based super-resolution (SR), where five state-of-the-art models—SRCNN, SRGAN, U-Net, SwinIR, and LTE—were tested against bicubic interpolation on a dataset of 888 panoramic radiographs downscaled by 4×. LTE outperformed all others, suggesting that SR models like LTE can meaningfully enhance radiographic resolution and image quality, thereby improving clinical interpretation and diagnostic precision (39). In terms of pathology detection, CNN-based U-Net architectures have been used to identify carious lesions from panoramic images within the DNS dataset, which includes 1,500 annotated images. Following expert review and data augmentation techniques such as flipping, mirroring, and rotation, U-Net, U-Net++, and U-Net3 + were trained and compared. U-Net3 + achieved the highest testing accuracy of 95%, attributed to its full-scale skip connections and deep supervision strategies, reinforcing its utility in early detection and automated classification of dental caries (29).

Collectively, these studies confirm that CNNs—and their advanced derivatives or hybrid integrations—represent some of the most effective and adaptable tools currently available for panoramic image analysis, excelling in both diagnostic accuracy and image quality enhancement.

### 3.4 AI performance in OPG analysis: evaluation of the supplementary aid in diagnosis

AI applications in OPG analysis have emerged over the past decade, building on advances in machine learning and medical imaging technologies. Early efforts focused on automating basic tasks such as tooth segmentation and numbering, demonstrating the feasibility of AI integration into dental radiography. With the development of more sophisticated deep learning algorithms—particularly CNNs—performance in detecting complex oral pathologies has significantly improved, enabling faster and more accurate preliminary assessments.

Evaluations of these AI tools have consistently shown promising results; however, they also reveal important limitations related to dataset diversity, annotation quality, and clinical generalizability. These challenges must be addressed to ensure safe and effective deployment alongside clinicians as a supplementary diagnostic aid. Results of recent studies evaluating AI performance in detecting various oral diseases and conditions are summarized in Table 1. Limitations and Challenges of AI use in dental imaging.

**Table 1:** Diagnostic performance of AI/ML-based systems applied to OPG analysis.

Author, Year	Nr. of test patients	Detection aims	Accuracy	Precision	Specificity	Fleiss kappa	F1 score
Kwiatek et al., 2025 (21)	55	probability of endodontic filling	90.72%	N/A	N/A	N/A	95.12%
Kwiatek et al., 2025 (21)	55	adequate obturation	55.81%	N/A	N/A	N/A	8.33%
Kwiatek et al., 2025 (21)	55	adequate density	62.75%	N/A	N/A	N/A	14.29%
Agrawal & Nikhade, 2022 (58)	127	caries detection	18.05%^a^	N/A	N/A	N/A	N/A
Agrawal & Nikhade, 2022 (58)	52	periapical lesions	88.2%	N/A	83.3%	0.83	N/A
Butnaru et al., 2025 (7)	Not specified	periodontal bone loss	N/A	N/A	N/A	0.76	N/A
Kazimierczak et al., 2024 (22)	55	dental anomalies	88%	N/A	85%	N/A	N/A
Kazimierczak et al., 2024 (22)	600	missing teeth	93.6%	N/A	96.4%	N/A	N/A
Kazimierczak et al., 2024 (22)	600	detecting supernumerary teeth	N/A	N/A	75.0%	N/A	N/A
Kazimierczak et al., 2024 (22)	600	impacted teeth	N/A	N/A	99.7%	N/A	N/A
Turosz et al., 2024 (23)	35	caries, fillings, missing teeth, plaque	>85%	N/A	>85%	N/A	N/A
Gaudin et al., 2024 (26)	348	osteoporosis related changes	92.6%	N/A	>91%	N/A	53%
Wu et al., 2025 (59)	400	apical position detection	96.6%	N/A	N/A	N/A	53%
Wu et al., 2025 (59)	400	sinus floor level identification	92.3%	N/A	N/A	N/A	
Alsolamy et al., 2025 (60)	135	incipient lesions	88.8%	84%	N/A	N/A	85.1%
AlGhaihab et al., 2025 (8)	100	stages of periodontitis	85.8%	N/A	N/A	N/A	N/A
Özçelik et al., 2024 (6)	313	stages of periodontitis	92.9%	72.4%	N/A	N/A	N/A
Mureșanu et al., 2024 (28)	1,628	periapical lesions, root fragments, impacted teeth, prosthetic elements – internal validation	>90%	N/A	N/A	N/A	N/A
Mureșanu et al., 2024 (28)	180	periapical lesions, root fragments, impacted teeth, prosthetic elements – external validation	N/A	N/A	N/A	N/A	N/A
Alharbi et al., 2023 (29)	4,296	dental cavities	95%	N/A	N/A	N/A	N/A
Ba-Hattab et al., 2023 (20)	713	periapical lesions	84.6%	N/A	85.6%	N/A	N/A
Rubiu et al., 2023 (61)	70	teeth identification via segmentation	100%	92.49%	N/A	N/A	N/A
Rohrer et al., 2022 (30)	1,781	identify dental restorations	N/A	N/A	N/A	N/A	95%
Rocha et al., 2022 (31)	1,500	identify dental restorations	96.59%	N/A	N/A	N/A	N/A
Prados-Privado et al., 2021 (32)	50	presence or absence of teeth	100%	N/A	N/A	N/A	N/A
Mohammed et al., 2024 (37)	1,200	skeletal growth maturation	98%	N/A	N/A	N/A	N/A
Küçük et al., 2025 (38)	407	impacted teeth	98.3%	N/A	N/A		96%
Akdoğan et al., 2025 (10)	2,000	difficulty of mandibular third molar extractions	N/A	97.00%	N/A	N/A	95.76%
Turosz et al., 2024 (23)	600	missing teeth, root canal fillings, implants	>85%	N/A	>85%	N/A	N/A
Turosz et al., 2024 (23)	600	endodontic lesions	N/A	N/A	N/A	N/A	65.30%

^a^ Caries detection depends on age; averaged value is given.

## 4 Limitations and challenges of AI use in dental imaging

### 4.1 Dependence on high-quality labeled datasets: AI performance is heavily reliant on the quality and quantity of training images

The effectiveness of AI in dental diagnostics is strongly dependent on the quality, quantity, and consistency of the training datasets (OPGs) used to develop and validate these systems. A study evaluating the diagnostic accuracy of an AI system in assessing periodontal parameters found that the model demonstrated superior consistency in detecting alveolar bone loss compared to human practitioners. Specifically, the AI achieved a mean score of 6.12 in identifying teeth with attachment loss, outperforming senior specialists (5.43), specialists (4.58), and general dentists (3.65), with statistically significant differences in maxillary arch detection. These findings illustrate AI's potential in periodontal diagnostics, particularly when supported by well-structured, high-quality training data (7). In pediatric dental panoramic radiographs, an AI algorithm demonstrated high specificity and accuracy (both above 85%) for detecting caries, fillings, and missing teeth, though with lower precision in identifying endodontic lesions and crowns, highlighting the need for larger and more diverse training datasets (23). A broad review of 55 peer-reviewed articles published between 2009 and 2022 on AI integration in dental specialties — spanning periodontology, endodontics, orthodontics, restorative dentistry, and oral pathology — confirmed that AI can enhance diagnosis, treatment planning, and risk prediction. Nonetheless, it emphasized the critical role of dataset quality, standardization, and data privacy, which remain major barriers to consistent and safe clinical adoption (40).

### 4.2 Algorithmic bias and generalization issues: AI models may yield variable results depending on the dataset used and may struggle to adapt to diverse populations

Although AI systems in dental diagnostics demonstrate strong potential, their performance often varies across conditions and datasets, raising concerns about algorithmic bias and limited generalizability. Pre-processing steps such as local contrast enhancement and noise reduction (e.g., contrast-limited adaptive histogram equalization and bilateral filtering), together with tiling strategies focusing on regions of interest, can partially mitigate these issues and have been shown to outperform models trained on full radiographs (28, 30). Segmentation approaches such as SAM2 (Figure 1) and deeper CNN architectures (Figure 2) enable the network to capture more complex anomalies, but may also increase the risk of overfitting or reduced transparency (11, 34–36, 45). Importantly, diagnostic bias is not limited to AI systems: Decoster et al. reported that radiographers rated 56% of radiographs as non-diagnostic or limited, compared to 30.7% for radiologists, highlighting the need for structured quality-assurance frameworks in both human and algorithmic decision-making (46).

### 4.3 Clinical acceptance of AI: the dental professionals perspectives in adopting AI

Artificial Intelligence is transforming oral healthcare and clinical applications, enhancing every aspect of dentistry today.

Clinically, AI improves treatment planning, refines periodontal assessments, and provides powerful diagnostic support that challenges traditional methods. Administratively, it automates charting, insurance attachments, and clinical documentation, saving time and reducing errors. AI systems in dentistry fall into four key categories: automated task performers, advisory tools, autonomous diagnostic engines, and integrated platforms combining clinical and operational intelligence.

AI software such as Second Opinion (Developed by Pearl Inc., Beverly Hills, CA, USA), Diagnocat (developed by Diagnocat Ltd., San Francisco, CA, USA), Automate (3Shape A/S, Copenhagen, Denmark) and Smilecloud (Smilecloud Biometrics, Timisoara, Romania) are already used by dental professionals for either automated diagnosis or treatment planning (41).

This shift redefines what's possible in dental care. Yet, as AI advances, we must prepare to responsibly manage its impact on clinical roles, patient trust, and healthcare equity.

Perceived medicolegal ambiguity, and trust-related barriers as significant obstacles to AI implementation in clinical workflows (27).Fears of professional autonomy loss, lack of integration in routines and clinical workflow or even loss of contact with the patients are identified as the main drivers in reluctance of AI acceptance. Proper training and education on the usage of AI are potential avenues to encourage users towards increasing AI adoption (42).

It was also reported that the diagnostic process in radiology is more established, compared to other disciplines (42). Studies performed in various countries confirm that the majority of dentist professionals consider AI as a supportive tool which could be adopted in practice (43–46).

However, effective AI implementation requires independent validation datasets and the use of standardized performance metrics, including sensitivity, specificity, precision, recall, AUC, and F1-score. Validated studies using deep learning models had also been pursued. One study had validated on CNNs tooth detection accuracies ranging from 91% to 98%, caries detection performance with F1-scores between 0.83 and 0.94, and segmentation Dice coefficients exceeding 0.90 (21, 23). Also, a CNN study validated periodontal bone loss detection with accuracies of 98% and F-score of 98.5% (47). Several models report validated ROC-AUC values above 0.95 for binary classification tasks in dental imaging (22, 23).

Next to the considerations pertaining to AI's integration in the clinical workflow, staff training or clinical human supervision, the enforcement of a regulatory framework for AI is required.

### 4.4 Ethical and regulatory considerations: the need for standards to ensure broad implementation of AI in dental imaging

A mixed-method study provides an in-depth analysis into the pressing ethical and regulatory challenges at the heart of AI implementation in dental imaging. Gathering insights from 102 diverse participants—including dentists, dental staff, AI developers, and patients—combined with in-depth interviews of key stakeholders, the research uncovers critical concerns shaping AI's clinical acceptance. Strikingly, 81.4% of respondents insist on obtaining explicit patient consent before any AI application, while 74.5% demand full transparency regarding how AI handles sensitive health data. These ethical apprehensions directly intersect with existing legal frameworks regulating AI in healthcare, highlighting a complex landscape where patient rights, data privacy, and technological innovation must be carefully balanced. This study not only maps key stakeholder perspectives, but also emphasizes the urgent need for coherent policies that safeguard trust as AI continues to reshape dental diagnostics (27).

The Food and Drug Administration (FDA) is one of the regulatory bodies discussed within the international frameworks for AI in healthcare (48). FDA has a risk-based strategy to oversee all of the medical devices, starting with the lowest risk in class I and ending with the highest risk in class III. Class III, involving the highest risk, requires premarket approval (49). Since 1996, patient privacy in the U.S. has been protected through the Health Insurance Portability and Accountability Act (HIPAA), which represents a key regulatory framework relevant to AI in healthcare (50). While the FDA ensures regulatory oversight, ethical review of research protocols involving human subjects is conducted by Institutional Review Boards (IRBs).

With the advent of new progress in Machine Learning (ML), in 2019 the FDA launched a Proposed Framework for Modifications to AI/ML-based Software as a Medical Device (SaMD). Based on stakeholders' feedback, in 2021 the AI/ML-based SaMD Action Plan was enacted to oversee the total product cycle of AI implementation in clinical settings. Two years later, a Predetermined Change Control Plan (PCCP) guidance was released, including the types of changes that can be implemented without requirements for additional regulatory review (49). The PCCP thus allows for a guided growth of the AI/ML which considers the evolutionary nature of the technology (50).

Considering the US's ethical and the regulatory framework, FDA is thus the agency tasked with the AI/ML-based SaMDs evaluation, approval and post-market monitoring (48).

**Figure 3:**
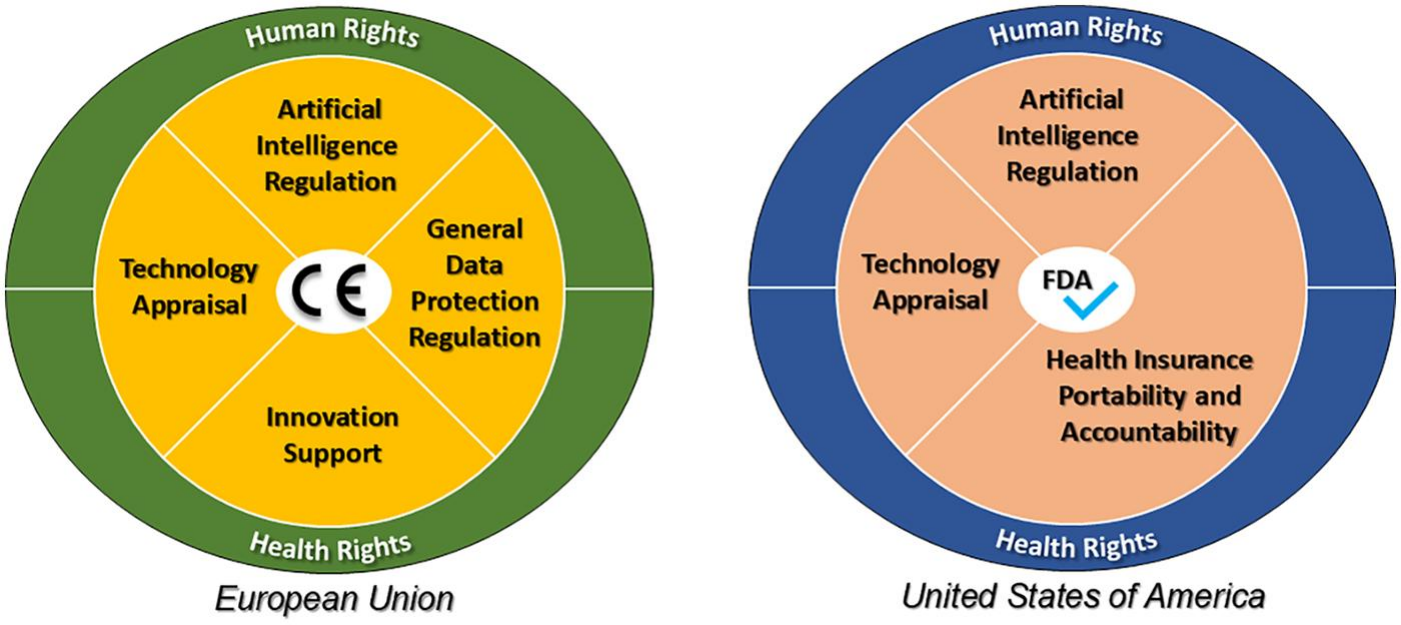
Regulatory framework required to obtain the CE marking in EU and the FDA approval in USA for the free trade of the AI/ML based SaMDs (51).

In the European Union (EU), the legal framework has important particularities compared to the US. The Treaty on the Functioning of the European Union (TFEU) defines, among others, (i) the competence of the EU to ensure the functioning of the single market for all EU countries and (ii) the objective of strengthening its scientific and technological bases (51). This allows the EU to set standards for the safety of products placed in the European Economic Area (EEA) and to provide funding for scientific innovation, such as through the Horizon Europe programs (51). The CE marking (see center of Figure 3) certifies that a product complies with all EU requirements and can be freely traded and used across the EEA.

The health and human rights policies in EU target standards that should benefit society and contribute to the well-being of individuals (51). The standards can be implemented either as a directive or as a regulation. A directive can be implemented through national law of each country, offering room for legal flexibility. One such directive is Directive (EU) 2022/2555, a cybersecurity policy for the protection of the EU against cyber-attacks which may target private medical data. Whilst a regulation applies directly to all of the EU member states on ratification. A relating regulation already enforced is the Cybersecurity Act Regulation (EU) 2019/881 which aims at creating common cybersecurity certification schemas and enables European Union Agency for Cybersecurity (ENISA) (51).

The EU equivalent of the FDA's HIPAA is Regulation (EU) 2016/679 which defines the General Data Protection Regulation (GDPR). However, the GDPR does not have a particular focus on health and medical data. GDPR rather deals with the processing of all of the individual's personal data by companies, indiscriminate of the company's activity domain (52).

The main regulatory body overseeing public health and medicinal products in the EU is the European Medicines Agency (EMA) (53). For medical devices, the regulatory framework is defined by the Medical Devices Regulation (EU) 2017/745 (MDR) and the *in vitro* Diagnostic Medical Devices Regulation (EU) 2017/746 (IVDR) (51). In May 2021, the risk classification of SaMDs based on diagnostic and therapeutic interactions was adopted within the MDR (53). According to the updated MDR, the majority of SaMDs are classified as high risk (class IIa or higher) (53, 56).

The EU Artificial Intelligence Act (AIA) was proposed in April 2021 and formally adopted in 2024. It addresses risk management, data governance, human oversight, transparency, accuracy, robustness, and cybersecurity, while defining the obligations of providers towards end users (53). The AIA also has direct implications for the regulation of AI/ML-based SaMDs within the EU's medical device framework. More broadly, the EU digital sector is most recently regulated by the Digital Markets Act (DMA) and the Digital Services Act (DSA), aimed at ensuring fair and open digital markets across the EEA (51).

As previously highlighted and synthesized in Figure 3, the EU combines multiple regulatory frameworks for AI/ML-based SaMDs. In order to simplify the evaluation processes of the high risk SaMDs for the EU market, the UE system relies on Notified Bodies to ensure compliance with the broad regulatory framework (48). Each EU country can designate within their jurisdiction conformity assessment bodies (i.e., Notified Bodies), and can carry their activities in other EU countries or non-EU countries.

Similar regulations which deal with the AI's use in healthcare settings were also adopted in other countries, such as United Kingdom, Australia, China or Singapore (53).

## 5 Concluding remarks

The integration of AI into dental imaging platforms marks a significant advancement in the standardization and efficiency of diagnostic workflows. Modern AI software embedded into everyday clinical tools has demonstrated the ability to reduce analysis time, enhance consistency, and assist clinicians in complex decision-making processes. These systems are not only automating image interpretation, but also supporting clinical documentation and treatment planning by providing structured and reproducible outputs (54). An important direction of current development, as highlighted by Patil et al. (55), is the use of real-time AI applications that analyze panoramic images during clinical encounters.

Can AI truly revolutionize dental imaging? This review challenges conventional perspectives on AI in dentistry by critically examining its transformative potential and real-world limitations. Far beyond mere technical innovation, today's deep learning advances — particularly in orthopantomography — promise to reshape how educators, clinicians, and students approach dental diagnostics. Yet, are we fully prepared for this paradigm shift? Drawing on recent breakthroughs, rigorous performance metrics, and front-line case studies, we dissected both the dazzling achievements and uncomfortable hurdles hindering AI's integration into everyday dental workflows, with special attention paid to the pressing regulatory, ethical, and technical dilemmas that demand urgent solutions. Where does promise end and reality begin? By mapping the shifting landscape of dental imaging, we invite educators, learners, and practitioners to rethink the future: Is AI the key to unlocking a new era in oral health, or are its risks and constraints still too great for widespread adoption? Finally, there is a critical need to develop AI systems with access to broader, more representative datasets to enhance generalizability and ensure equitable diagnostic accuracy across diverse patient populations.

But beyond the clinic, what might AI mean for the dental classroom and preclinical training? Could algorithms that segment anatomy or detect pathologies in real time become dynamic teaching aids — accelerating the mastery of radiographic interpretation, exposing learners to rare cases, and even personalizing feedback at a scale no educator alone could provide? If AI systems begin to shape how tomorrow's dentists learn to see, diagnose, and plan, are we prepared to rethink traditional pedagogical models? These questions press us to view AI not only as a clinical tool, but also as a transformative force in preparing the next generation of dental professionals. The echo of such questions provides the fertile ground of new undergraduate curriculum prospects (56, 57).
